# Investigating resistin like beta (RETNLB) as a tumor promoter for oral squamous cell carcinoma

**DOI:** 10.1186/s13005-021-00272-4

**Published:** 2021-06-22

**Authors:** Hong Jin, Hui Miao, Yuan-Wen Nie, Yang-Yang Lin

**Affiliations:** 1 College of Stomatology, Mudanjiang Medical University, Mudanjiang, 157000 Heilongjiang China; 2 Department of Gynecology and Obstetrics, the Second Affiliated Hospital of Mudanjiang Medical University, Mudanjiang, 157000 Heilongjiang China; 3 Department of Hepatobiliary Surgery, the Second Affiliated Hospital of Mudanjiang Medical University, Mudanjiang, 157000 Heilongjiang China; 4 Department of Stomatology, Hongqi Hospital Affiliated to Mudanjiang Medical University, No.708 of Guanghua Street, Mudanjiang, 157000 Heilongjiang China

**Keywords:** RETNLB, Oral squamous cell carcinoma, Proliferation, Invasion and migration, TLR/2/4/ERK pathway

## Abstract

**Background:**

Oral cavity cancer ranks the sixth most common malignancy worldwide, of which oral squamous cell carcinoma is the predominant type. This study aimed to investigate the function and the underlying mechanism of resistin like beta (RETNLB) in oral squamous cell carcinoma.

**Methods:**

The data of oral squamous cell carcinoma samples from The Cancer Genome Atlas database was used to examine RETNLB expression and assess its correlation with the clinical outcomes. Biological functions of RETNLB on the growth, invasion and migration of cells were determined by cell counting kit 8, clonogenic growth, and Transwell assays. Gene set enrichment analysis was utilized to identify the important gene sets associated with RETNLB expression, which was further confirmed by western blot.

**Results:**

We found that RETNLB was upregulated in oral squamous cell carcinoma tissues and cells. High expression of RETNLB was closely linked to age and pathological tumor, and significantly related to poor survival of oral squamous cell carcinoma patients. Further functional experiments showed that knockdown of RETNLB significantly reduced the viability, mobility and invasiveness of cells. Moreover, gene set enrichment analysis suggested that Toll-like receptor signaling pathway was significantly correlated with high RETNLB expression. Further western blot analysis verified that silencing RETNLB could notably suppress the protein levels of Toll-like receptor 2, Toll-like receptor 4 and phosphor- extracellular signal-regulated kinase.

**Conclusions:**

These results suggested that downregulation of RETNLB may restrain the progression of oral squamous cell carcinoma by inactivating TLR/2/4/ERK pathway.

## Introduction

Oral cavity cancer is a part of head and neck cancer, which ranks the sixth most common malignancy worldwide [1]. Oral cavity cancer accounts for approximately 3% of all malignant tumors [2]. Clinically, oral squamous cell carcinoma is the predominant type of oral cavity cancer, accounting for 90% [3]. At present, the treatments of oral squamous cell carcinoma mainly include, radiotherapy, chemotherapy, and surgical resection or a combination of these three methods. Although therapeutic methods are constantly improving, in fact, there exist several risks and life quality is reduced after treatment [4]. In addition, the abundance of blood vessels and nerves in maxillofacial tissue leads to a high probability of lymph node metastasis and recurrence for oral squamous cell carcinoma patients [5]. As a result, the 5-year survival rate has not increased significantly in recent year and has been maintained at 50–60%, while it is worse in patients with advanced stage and recurrence [6]. Therefore, the exploration of biomarkers and therapeutic targets for diagnosis, prognosis and treatment of oral squamous cell carcinoma is one of the urgent problems to be solved in clinical and basic research.

The resistin like molecules (RELMs) are a family of mammalian secreted proteins, consisting of 105 to 138 amino acids [7]. So far, this family contains four proteins, RELMα/RETNLA, RELMβ/RETNLB, Resistin/RETN and RELMγ/RETNLG, which were discovered in different disease settings less than 20 years ago, leading to different nomenclature [8, 9]. Recently, in addition to being implicated in microbial infections, inflammatory disease, and metabolic disorders, some of these molecules have also been reported to be involved in the progression of certain cancers [7, 10–12]. For example, RETN, as a pro-inflammatory cytokine, was found to bind to TLR4 on the cell membrane of colon cancer, initiating Toll-like receptor 4-myeloid differentiation primary response gene 88-dependent activation of ERK [13]. Interestingly, we found that no report reported the RETNLA expression or function in cancers, but as early as a decade ago, positive RETNLB expression had been tested in 81.25% of 80 colon cancer patients, where there was a positively correlation between RETNLB expression and survival time [14]. Similarly, 65.4% of 136 human gastric carcinoma patients were positive for RETNLB expression, which led to a significantly longer overall survival than patients with negative RETNLB expression [15]. Besides, functional experiments demonstrated that RETNLB-overexpression could remarkably enhance the invasiveness and mobility of gastric carcinoma cells and promote the progression of epithelial-mesenchymal transition [16]. These evidences suggest that RETNLB is expected to be a potential biomarker in some tumors, and led us to suspect that RETNLB may also play a role in oral squamous cell carcinoma, which has not yet been reported.

In the present study, we investigated the expression and prognostic value of RETNLB in oral squamous cell carcinoma patients through bioinformatics analysis of the data from The Cancer Genome Atlas database. Moreover, the effect of RETNLB on the growth, migration and invasion of oral squamous cell carcinoma cells as well as the underlying mechanism were explored through biological experiments.

## Materials and methods

### Public database-based analysis

We downloaded the expression data and clinical data of head and neck squamous cell carcinoma dataset (TCGA-HNSC) from The Cancer Genome Atlas (TCGA, https://cancergenome.nih.gov) database. Then the data of oral squamous cell carcinoma-related tumor (including lip, palate, tongue, base of tongue, floor of mouth, gum and oropharynx) samples and adjacent non-tumor tissues were screened from TCGA-HNSC dataset. Finally, 338 oral squamous cell carcinoma samples (tumor) and 32 adjacent non-tumor tissue samples were selected for analyzing the expression of RETNLB. Among the 338 oral squamous cell carcinoma samples, 266 samples possess complete clinical data were employed for assessing the prognostic values. The Kaplan-Meier method was utilized to plot survival curve and the log-rank test was used to estimate survival differences between high and low RETNLB expression groups. The relationship between RETNLB level and clinical parameters was evaluated by chi-square test. Gene set enrichment analysis was conducted using 3.0 version (http://www.broadinstitute.org/gsea/) to identify RETNLB related gene sets.

### Cancer cell line culture

One human oral epithelial cell line and two human oral squamous cell carcinoma cell lines CAL27 and TCA-83 were all acquired from Chinese Academy of Sciences (Shanghai, China). These three cells were incubated in Dulbecco’s modified Eagle’s medium (Gibco, USA) containing 10% fetal bovine serum (ExCell Bio, China) and 100 μg/mL streptomycin/penicillin in an incubator with a 5% CO_2_ atmosphere at 37 °C.

### Transient transfection

Two sequences of small interference RNA (siRNA) targeting to the RETNLB (si-RETNLB#1 5′-GGTTGTCACTGGATGTGCTTG-3′; si-RETNLB#2 5′-CAGTCGTCAAGAGCCTAAGAC-3′) were synthesized to silence the expression of RETNLB. The sequence 5′-AATTCTCCGAACGTGTCACGT-3′ was designed as the negative control siRNA (si-NC), without homologous sequence of RETNLB. All siRNAs were designed by Genepharma Co., Ltd. (Shanghai, China). Transfection of siRNAs was conducted using the Lipofectamine 3000 reagent (Thermo Fisher Scientific, Carlsbad, USA) according to manufacturers’ protocol. After transfection for 48 h, the cells were collected to detect the transfection efficiency and used to perform the following functional experiments.

### Real-time quantitative polymerase chain reaction analysis

Total RNA was extracted from transfected cells with Trizol reagent (Thermo Fisher Scientific). PrimeScript RT Reagent Kit was used to reverse transcribe mRNA into cDNA. Real-time qPCR was carried out using the SYBR Green Mix kit (Thermo Fisher Scientific) in an Applied Biosystem 7500 real-time qPCR system (Foster City, CA, USA). The mRNA levels of RETNLB were normalized to GAPDH and analyzed using the 2^−ΔΔCq^ method. Primers used for qPCR are as follows:

RETNLB forward: 5′-GCAAGAAGCTCTCGTGTGCTAG-3′,

RETNLB reverse: 5′-AACATCCCACGAACCACAGCCA-3′;

GAPDH forward: 5′-GTCTCCTCTGACTTCAACAGCG-3′,

GAPDH reverse: 5′-ACCACCCTGTTGCTGTAGCCAA-3′.

### Western blot assay

Total proteins were isolated from transfected cells by radioimmunoprecipitation assay lysis buffer (Beyotime, Nantong, China) containing protease inhibitors. The protein concentration was measured by a bicinchoninic acid assay (Thermo Fisher Scientific). Following separation of the extracted proteins (20 μg/well) by 10% sodium dodecyl sulfate-polyacrylamide gel electrophoresis, the proteins were transferred onto polyvinylidene fluoride membranes. The membranes were then blocked using 5% non-fat dry milk for an hour, and incubated with primary antibodies at 4 °C overnight. Next, the membranes were rinsed with Tris Buffered Saline Tween thrice, followed by an hour of incubation with the appropriate secondary antibodies. Antibodies used in this study are listed as follows: anti-RETNLB (1:1000, ab271225), anti-TLR2 (1:1000, ab213676), anti-TLR4 (1:1000, ab13556), anti-phosphor (p)-ERK (1:1000, ab214036), anti-ERK (1:1000, ab17942), and anti-GAPDH (1:1000, ab37168). All the antibodies were obtained from Abcam (Cambridge, UK). After developing with an enhanced chemiluminescence detection system (Thermo Fisher Scientific), the Bio-Rad image analysis system (Hercules, CA, USA) was used to capture images. GAPDH was served as internal reference. Relative gray values were quantified by the ImageJ software 1.44 (National Institutes of Health, Bethesda, MD, USA).

### Cell proliferation assay

Cell counting kit 8 was utilized to determine the proliferation of transfected cells. Following the manufacturers’ protocol, 1 × 10^3^ cells/well were plated in 96-well plates and cultivated for 24, 48, 72 h of independent time period. On respective day, 10 μL of cell counting kit 8 reagent was directly added to each well. Upon incubation for 2 h, the optical density was obtained at 450 nm with a BioTeK Synergy H1 plate reader (Winooski, VT).

### Clonogenic growth assay

Clonogenic growth assay was conducted to determine the effect of RETNLB on colony-forming abilities of oral squamous cell carcinoma cells. After transfection for 48 h, 400 cells of each group were seeded into the 60 mm dishes which contained 5 mL Dulbecco’s modified Eagle’s medium with 10% fetal bovine serum, and incubated at 37 °C under 5% CO_2_ atmosphere for 12 days until colonies were visible. Next, the colonies were rinsed with phosphate buffered saline twice, fixed with 4% paraformaldehyde for 30 min and stained with 0.1% crystal violet for 15 min, after which they were photographed and counted manually.

### Transwell assay

Transwell chambers with 8 μm of pore size were used to evaluate invasive and migratory abilities of transfected cells. Matrigel matrix (Sigma, USA) diluted with serum-free medium (1:6) was precoated in upper chambers for invasion assay. Matrigel was not required for migration assay. Transfected cells were trypsinized and resuspended into a single-cell suspension. The cell suspension (100 μL) was added into the upper chamber and 500 μL of medium with 10% fetal bovine serum was supplied to the lower chamber. After being incubated at 37 °C for 24 h, the cells migrated/invaded to the underside of the membrane were rinsed, fixed, and stained, after which they were imaged and counted under an inverted microscope.

### Data analysis

Statistical analysis was conducted using the GraphPad Prism 6.0 software (La Jolla, CA, USA) and the SPSS 22.0 software (Armonk, NY, USA). Measurement data are expressed as mean ± standard deviation for at least triplicate experiments. A two-tailed *t* test was applied for double-group comparison and one-way analysis of variance was adopted for multiple-group comparison, followed by post hoc Dunnett’s test. *p* < 0.05 was defined to be indicative of a statistically significant difference.

## Results

### RETNLB is upregulated in oral squamous cell carcinoma and involved in poor outcomes

**Fig. 1:**
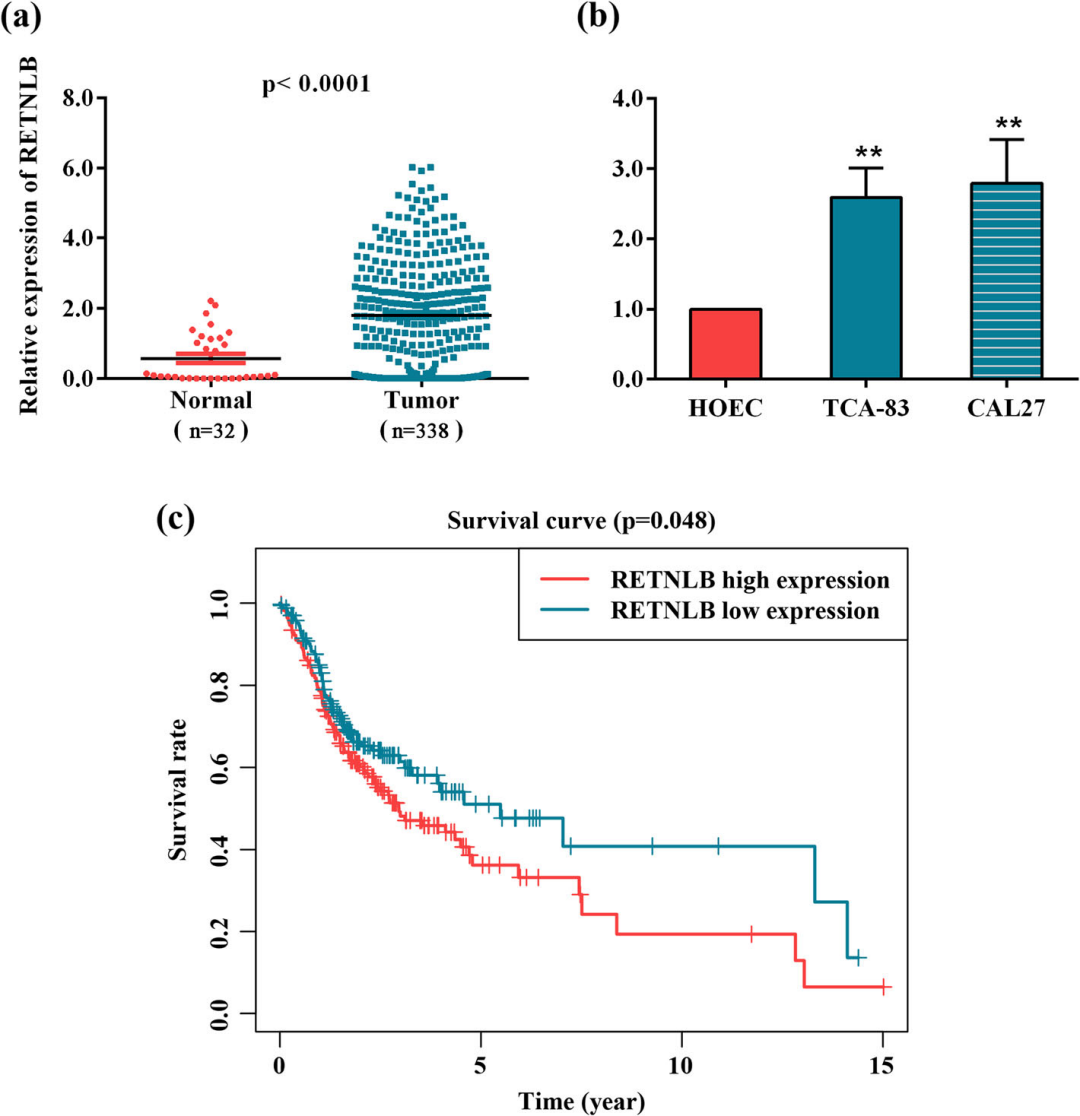
Expression of RETNLB and survival curve. (**a**) Expression of RETNLB in oral squamous cell carcinoma tissues and the adjacent non-tumor tissues was evaluated according to The Cancer Genome Atlas database. (**b**) Expression of RETNLB in oral squamous cell carcinoma cell lines (TCA-83 and CAL27) and normal cell line HOEC was identified by real-time qPCR. **p < 0.01 vs. HOEC group. Data are presented as means ± standard deviation. Columns, means. Bars, standard deviations. (**c**) Overall survival curve of oral squamous cell carcinoma patients with high and low RETNLB expression was plotted by Kaplan-Meier method. High RETNLB expression vs. low RETNLB expression. The median overall survival and 95% confidence interval (CI) of RETNLB low expression group were 7.041 and 2.047–12.035, respectively. The median overall survival and 95% CI of RETNLB high expression group were 2.956 and 2.042–3.870, respectively

To illuminate the expression of RETNLB in oral squamous cell carcinoma, the levels of RETNLB were firstly examined in 338 oral squamous cell carcinoma tissues and 32 adjacent non-tumor tissues based on the The Cancer Genome Atlas database. Result shown in Fig. 1a showed that RETNLB was upregulated in oral squamous cell carcinoma tissues compared with para-tumor samples. Consistently, RETNLB was observed to be overexpressed in two commonly used oral squamous cell carcinoma cell lines in comparison with non-tumor cell line (*p* < 0.01, Fig. 1b), suggesting that RETNLB might play a carcinogenic effect in oral squamous cell carcinoma.

We further assessed the correlation between RETNLB and the clinical outcomes in patients with oral squamous cell carcinoma. As shown in Fig. 1c, patients showing low RETNLB expression had a notably longer overall survival than those with high RETNLB expression (*p* < 0.05). The correlation between RETNLB expression and clinical features is summarized in Table 1. The RETNLB expression was not affected by gender, or by grade, clinical stage and pathological node (*p* > 0.05). Of note, in the oral squamous cell carcinoma cases examined, the expression of RETNLB was found to be positively correlated with age and pathological tumor (*p* < 0.05). Together, all these data demonstrated that RETNLB may have a prognostic value in oral squamous cell carcinoma.

**Table 1:** Correlation between RETNLB expression and clinical features

Characteristics	Expression of RETNLB		***P*** value
	Low	High	
Age			0.013*
< 60	67	47	
≥ 60	66	86	
Gender			0.285
female	36	44	
male	97	89	
Grade (G)			0.874
G1 + G2	108	109	
G3 + G4	25	24	
Stage			0.184
I + II	34	25	
III + IV	99	108	
Tumor (T)			0.017*
T1 + T2	62	43	
T3 + T4	71	90	
Node (N)			0.323
N0	55	63	
N1 + N2	78	70	

^*^P < 0.05

### RETNLB knockdown restrains the growth of oral squamous cell carcinoma cells

**Fig. 2:**
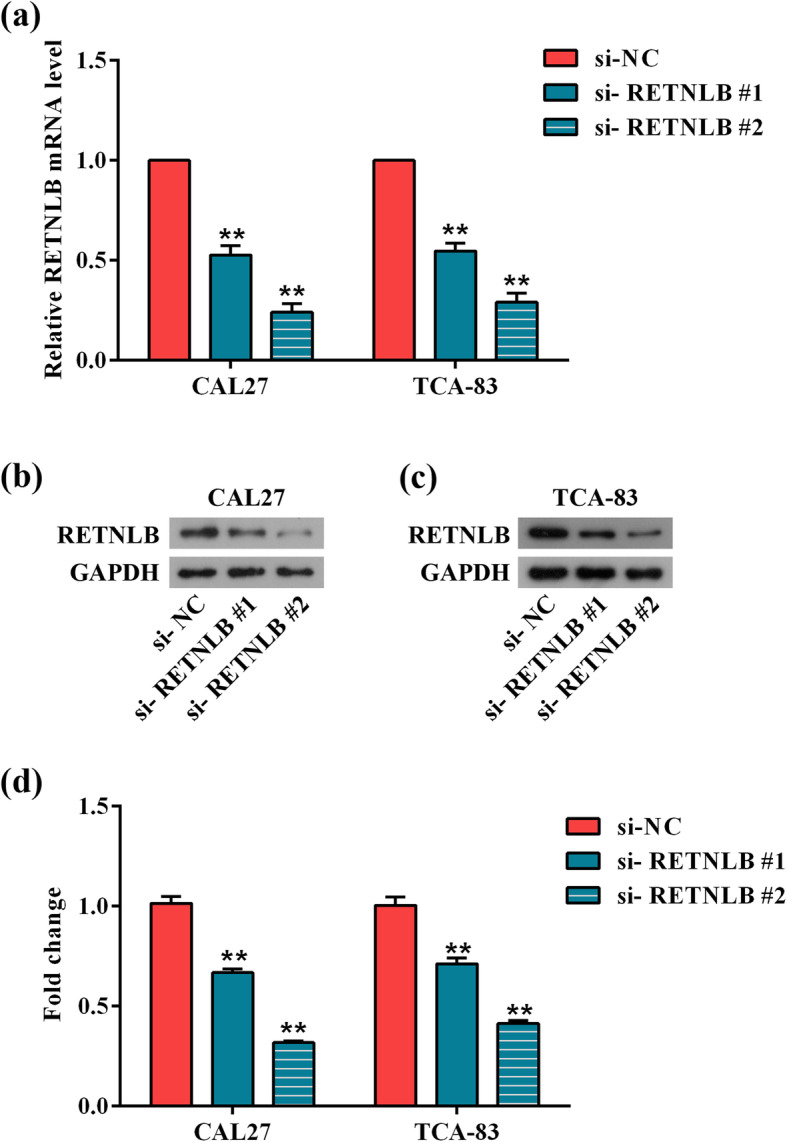
Detection of transient transfection efficiency of siRNAs. (**a**) Relative mRNA expression levels of RETNLB in CAL27 and TCA-83 cells were detected by real-time qPCR after transfected with si-NC, si-RETNLB#1 and si-RETNLB#2. (**b**, **c**) Representative images of western blot. Relative protein levels of RETNLB in CAL27 (**b**) and TCA-83 (**c**) cells were measured after transfected with si-NC, si-RETNLB#1 and si-RETNLB#2. (**d**) Protein expression histogram of RETNLB in CAL27 and TCA-83 cells. Data are presented as means ± standard deviation. Columns, means. Bars, standard deviations. **p < 0.01 vs. si-NC group

Before elucidating the functional role of RETNLB in oral squamous cell carcinoma, CAL27 and TCA-83 cells were transfected with si-RETNLB#1 and si-RETNLB#2 to down-regulate the expression of RETNLB. In comparison with si-NC group, the mRNA and protein levels of RETNLB were remarkably reduced in si-RETNLB-transfected cells (*p* < 0.01, Fig. 2a-d). Noticeably, the levels of RETNLB were lower in si-RETNLB#2 group than that in si-RETNLB#1 group, thus si-RETNLB#2 was chose to perform the next functional experiments, and expressed as si-RETNLB.

**Fig. 3:**
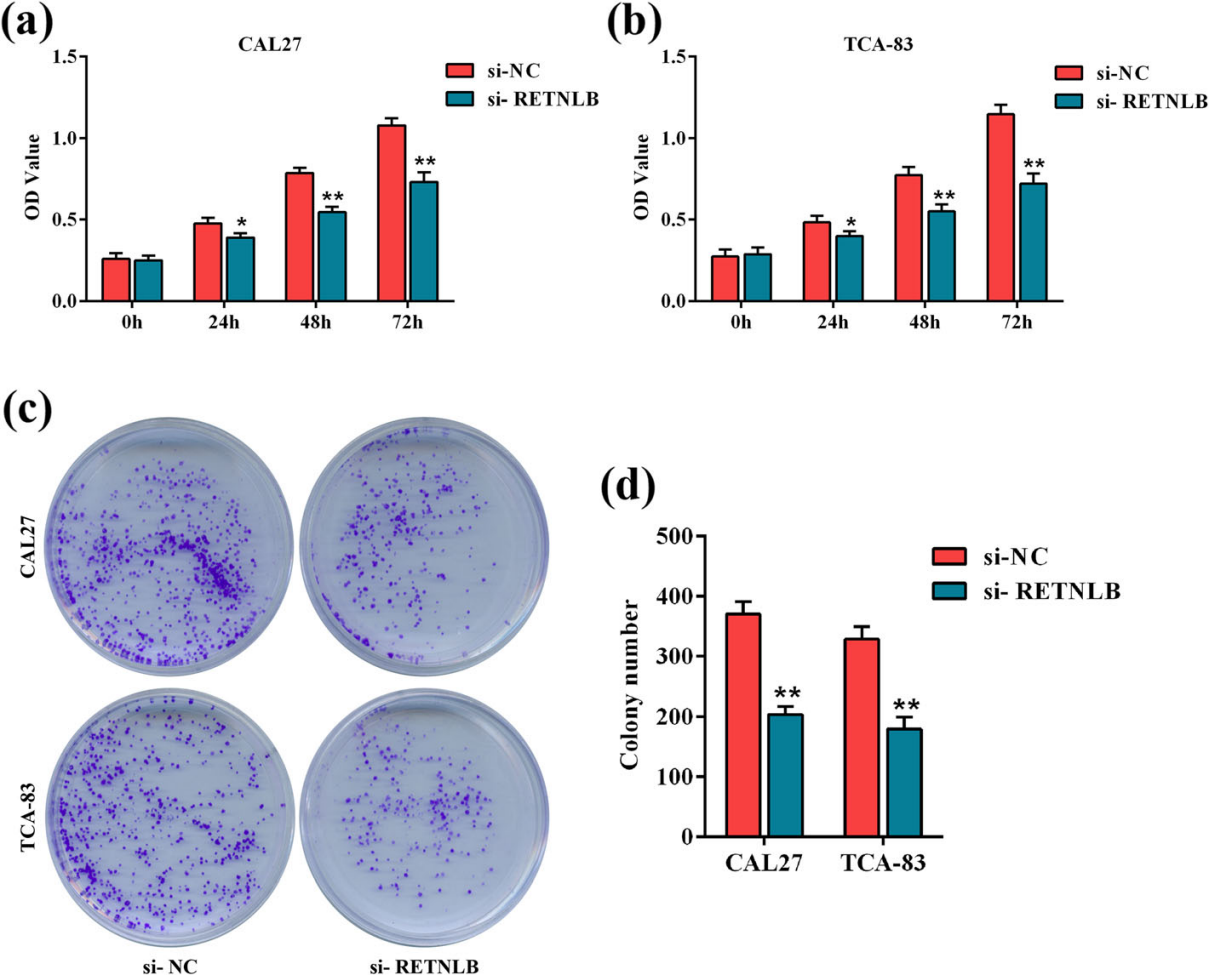
The effect of RETNLB knockdown on the growth of oral squamous cell carcinoma cells. RETNLB knockdown on the optical density values of CAL27 (**a**) and TCA-83 (**b**) cells were determined by cell counting kit 8 assay. (**c**, **d**) RETNLB knockdown on the colony-forming capacities of CAL27 and TCA-83 cells were assessed by clonogenic growth assay. (**c**) Representative clone pictures. (**d**) The number of colonies was routinely calculated. Data are presented as means ± standard deviation. Columns, means. Bars, standard deviations. *p < 0.05 vs. si-NC group. **p < 0.01 vs. si-NC group

To elaborate the function of RETNLB in the viability of oral squamous cell carcinoma cells, cell counting kit 8 and clonogenic growth assays were conducted. Compared with the corresponding si-NC group, the OD values of si-RETNLB-transfected CAL27 and TCA-83 cells were significantly reduced at 24, 48 and 72 h (*p* < 0.05, Fig. 3a). Analogously, the numbers of colonies of si-RETNLB-transfected CAL27 and TCA-83 cells were significantly less than si-NC-transfected cells, suggesting that RETNLB knockdown reduced the colony-forming capacities of oral squamous cell carcinoma cells. Collectively, the data indicated that downregulation of RETNLB may suppress the tumor growth in oral squamous cell carcinoma.

### Silencing RETNLB suppresses mobility and invasiveness of oral squamous cell carcinoma cells

**Fig. 4:**
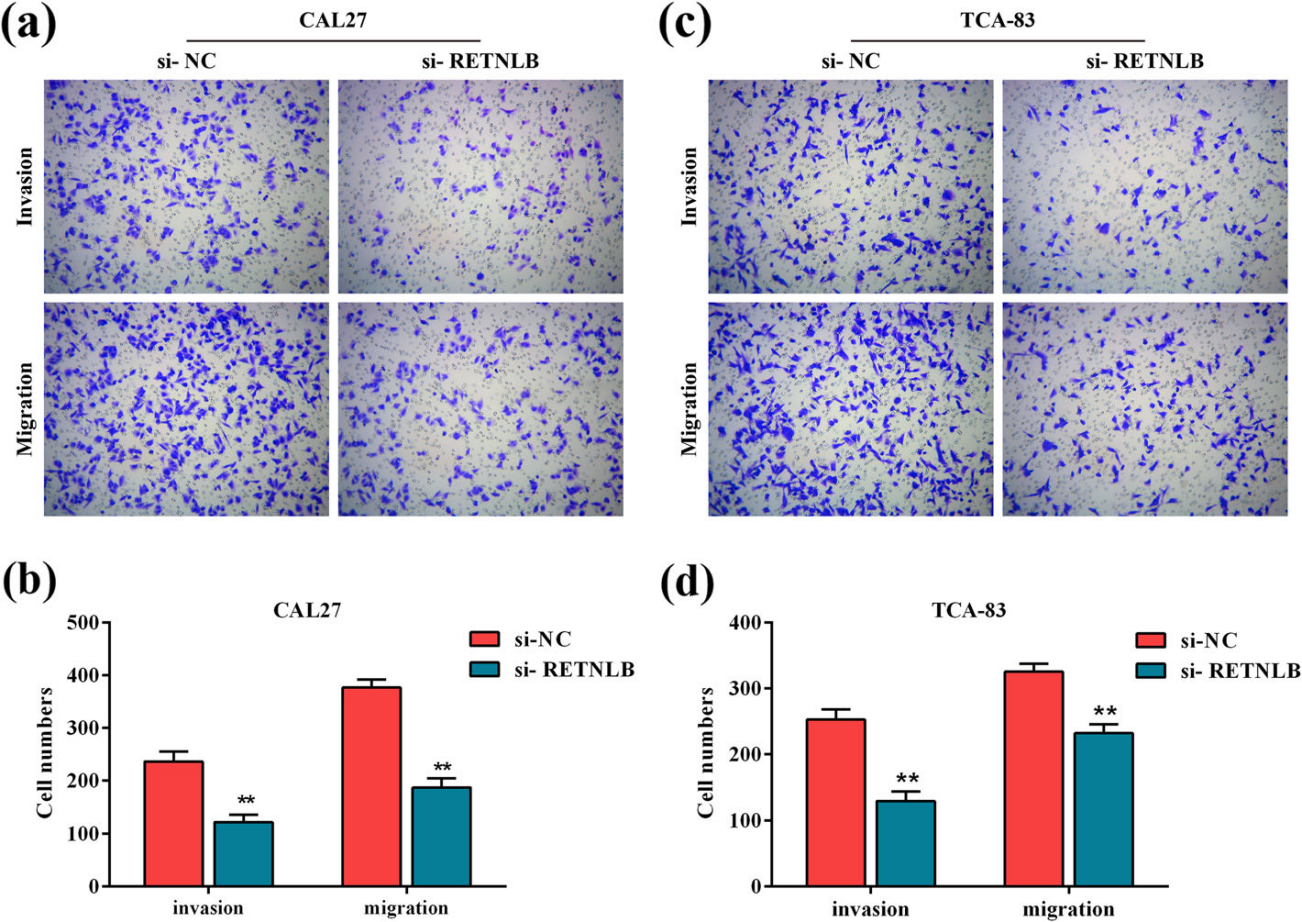
The effect of RETNLB knockdown on the invasion and migration of oral squamous cell carcinoma cells. Transwell assays were used to determine RETNLB knockdown on the mobility and invasiveness of CAL27 (**a**, **b**) and TCA-83 (**c**, **d**) cells. (**a**, **b**) Representative pictures. (**b**, **d**) The number of invaded/migrated CAL27 (**b**) and TCA-83 (**d**) cells was counted. Data are presented as means ± standard deviation. Columns, means. Bars, standard deviations. **p < 0.01 vs. si-NC group

Transwell assay was implemented to explore the effect of RETNLB on the mobility and invasiveness of oral squamous cell carcinoma cells. Numbers of invaded CAL27 and TCA-83 cells visibly decreased in si-RETNLB groups compared with their corresponding si-NC groups (*p* < 0.01, Fig. 4a-d). Consistently, transfection of si-RETNLB resulted in a significant reduction in the numbers of migrated CAL27 and TCA-83 cells compared with si-NC (*p* < 0.01, Fig. 4a-d). Altogether, these findings showed that silencing RETNLB inhibited the aggressive behaviors of oral squamous cell carcinoma cells in vitro.

### Downregulation of RETNLB inhibits oral squamous cell carcinoma progression through the TLR2/4/ERK signaling pathway

**Fig. 5:**
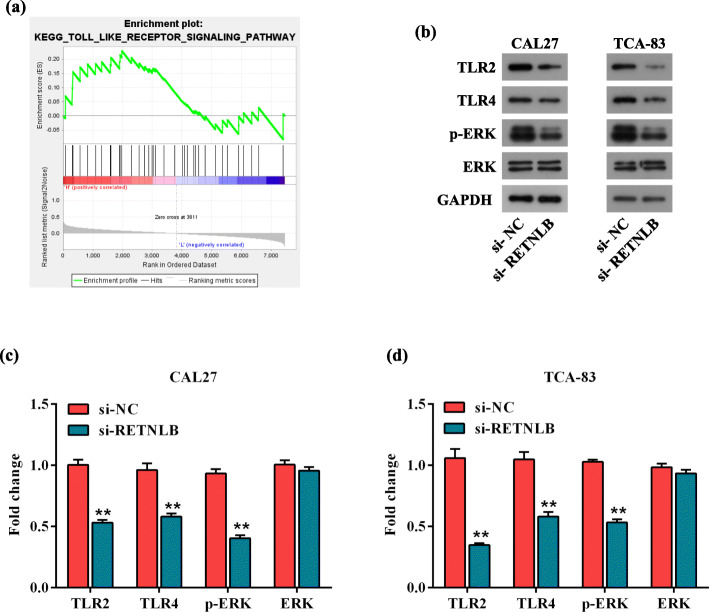
The effect of RETNLB knockdown on the TLR2/4/ERK signaling pathway-related markers. (**a**) Gene set enrichment analysis showed the enrichment of the TLR signature in the high RETNLB expression group according to the RNA-Seq dataset from the The Cancer Genome Atlas. (**b**, **c**, **d**) The protein levels of TLR2, TLR4, p-ERK, and ERK in CAL27 (**b**, **c**) and TCA-83 (**b**, **d**) cells were determined by western blot after transfected with si-NC and si-RETNLB. (**b**) Images of representative protein bands. (**c**, **d**) Protein expression histogram of markers in CAL27 (**c**) and TCA-83 (**d**) cells. Data are presented as means ± standard deviation. Columns, means. Bars, standard deviations. **p < 0.01 vs. si-NC group

To gain insight into RETNLB-mediated molecular pathways in oral squamous cell carcinoma, gene set enrichment analysis was conducted using the RNA-Seq dataset from The Cancer Genome Atlas database, which contains 338 oral squamous cell carcinoma tissues samples. The samples were divided into high and low RETNLB groups on the basis of the median RETNLB expression. Gene set enrichment analysis showed that RETNLB had a positive correlation with TLR signature (Fig. 5a). To our knowledge, nearly all TLRs (except TLR3) induce key pathways in various immune cells that is myeloid differentiation primary response gene 88-dependent, involving phosphorylation of mitogen activated kinase-like protein (MAPK), then translocating to the nucleus to activate transcription factors [17, 18]. Since the ERK-related intracellular signaling pathway is known as the classic of MAPK signaling, we speculated that the expression of RETNLB might be correlated with the activation of TLR/ERK signaling pathway. Through western blot, we found that the protein levels of TLR2, TLR4 and p-ERK were decreased in both CAL27 and TCA-83 cells by silencing RETNLB (*p* < 0.01, Fig. 5b-d). Interestingly, no significant change in the ERK level was observed after depletion of RETNLB (*p* > 0.05). These detections demonstrated that downregulation of RETNLB suppresses the progression of oral squamous cell carcinoma cells might by inactivation of the TLR2/4/ERK pathway.

## Discussion

The occurrence and development of oral squamous cell carcinoma is the common result of multiple genes and factors, however, the specific mechanism is still unclear. Currently, there are no clinically specific and sensitive markers for oral squamous cell carcinoma. Thus, new prognostic tumor markers need to be identified to provide more effective therapeutic targets for oral squamous cell carcinoma. Herein, we illustrated that RETNLB was up-regulated in oral squamous cell carcinoma and negatively correlated with the overall survival of patients with oral squamous cell carcinoma. Furthermore, we revealed that RETNLB acted a promoting role in the malignant development of oral squamous cell carcinoma cells via regulating TLR2/4/ERK pathway. These results highlighted the critical role of RETNLB and suggested it as a considerable biomarker for the treatment of oral squamous cell carcinoma.

RETNLB is an intestinal goblet cell-specific protein and is notably upregulated during intestinal inflammation [19]. Initially, RETNLB is characterized as hormones that modulate insulin action [20]. However, with the in-depth studies, subsequent reports demonstrated that RETNLB also plays a role in several research areas, such as inflammatory disease [21], cancer [16], and metabolic function [22]. In tumors, previous reports have suggested that positive expression of RETNLB were detected in most tissues from gastric carcinoma and colon cancer patients [14, 15], suggesting that the dysregulation of RETNLB may be valuable for the diagnosis of some cancers. In the present study, the abnormal expression of RETNLB was also found in 338 oral squamous cell carcinoma tissues compared to paratumor tissues based on data from The Cancer Genome Atlas, hinting its involvement in oral squamous cell carcinoma. The evidences supporting this view are that the overall survival rate of patients with low RETNLB expression was significantly longer than that of high RETNLB expression patients, and RETNLB was found to be associated with pathological tumor and age. What’s more, RETNLB revealed a high prognostic performance in colorectal cancer, and was further clarified to be correlated with pathological metastasis and vital status [23]. Additionally, RETNLB positivity in colon cancer was observed to be associated with lymph node metastasis and histological grade of differentiation, and led to a notably longer postoperative survival time [14]. All these findings support that RETNLB might be a valuable prognostic biomarker in oral squamous cell carcinoma patients, and implying that further exploration of RETNLB’s role in oral squamous cell carcinoma is necessary.

Although most studies on RETNLB have focused on its role in intestinal defense against parasitic infections and inflammation of the colon, its role in tumor biological functions is receiving increasing attentions [24]. Previous detections have revealed that RETNLB-overexpression can promote the gastric carcinoma cells’ migration and invasion and facilitate the progression of epithelial-mesenchymal transition [16]. Moreover, reduced RETNLB level has been shown to suppress the formation of abdominal aortic aneurysm [25]. Inspired by these findings, a series of functional tests were performed to illuminate the biological role of RETNLB in oral squamous cell carcinoma. The data suggested that depletion of RETNLB exhibited an inhibitory effect on the cells growth, invasion and migration, which provided a basis for demonstrating that targeting RETNLB may restrain the progression of oral squamous cell carcinoma.

To help illuminate the depth mechanisms by which RETNLB facilitates the progression of oral squamous cell carcinoma, gene set enrichment analysis was conducted. The data surprisingly showed that high RETNLB expression was positively linked to the TLR signaling pathway. As we know, TLRs are transmembrane proteins expressed by chronic inflammatory cells and endothelial cells during inflammation, in response to microbial products [26]. The TLR family is a large family with many members. One of them, TLR2, was found to be at great risk in oral squamous cell carcinoma, as evidenced by the high mRNA expression in 5/6 oral squamous cell carcinoma cell lines [18]. Moreover, previous investigations revealed that TLR4 was functionally expressed in oral squamous cell carcinoma cells, and high level of TLR4 was linked to a short survival rate [27, 28]. Thus, western blot assay was used to verify our suspicion that RETNLB played the tumor-promoting effect in oral squamous cell carcinoma cells through regulating the TLR2 and TLR4. As expected, the protein levels of TLR2/4 were significantly reduced after RETNLB deficiency. Furthermore, by consulting literatures, RETNLB was reported to regulate proliferation of human diabetic nephropathy mesangial cells by MAPK signaling pathway, and has the potential to be a mediator to contribute to airway remodeling at least partly via MAPK signaling pathway [29], which encouraged us to explore the correlation between RETNLB and MAPK pathway. Our result proved that silencing RETNLB reduced the phosphorylation of ERK without affecting the expression of ERK. Unfortunately, using the gene set enrichment analysis, no remarkable correlation was observed between RETNLB expression and MAPK signature, possibly due to the lack of relevant information in the public dataset. So, in our future studies, it is necessary to establish our own clinical dataset to further verify these results. Collectively, these evidences demonstrate that RETNLB deficiency suppresses the viability, mobility and invasiveness of oral squamous cell carcinoma cells partly by inactivating the TLR2/4/ERK signaling pathway.

The weaknesses of the present study must be pointed out. The biological role of RETNLB was only explored in oral squamous cell carcinoma cells, further animal experiments were required for verification. Furthermore, clinical samples collected by ourselves are needed for confirming the results obtained from public database. Nevertheless, the results in two cell lines and the bioinformatics analysis based on the public database unanimously illustrating the positive role of RETNLB in oral squamous cell carcinoma cell malignant development and the predictive potential on prognosis of oral squamous cell carcinoma patients.

## Conclusion

In conclusion, we described that RETNLB was upregulated in oral squamous cell carcinoma and led to a worse survival. Moreover, functional experiments verified that the down-regulation of RETNLB inhibited the growth and movement-related phenotypes of oral squamous cell carcinoma cells. Mechanistically, RETNLB possibly affects the phenotypes of oral squamous cell carcinoma cells partly by modulating the TLR2/4/ERK signaling pathway. All these data suggested that RETNLB may be a novel bio-target for the therapeutic intervention of oral squamous cell carcinoma.

## Data Availability

The data were available from the corresponding author on reasonable request.
